# Applicability of minimally invasive surgery for clinically T4 colorectal cancer

**DOI:** 10.1038/s41598-020-77317-2

**Published:** 2020-11-23

**Authors:** Yu-Tso Liao, Jin-Tung Liang

**Affiliations:** 1 Graduate Institute of Clinical Medicine, College of Medicine, National Taiwan University, Taipei, Taiwan, ROC; 2 Division of Colorectal Surgery, Department of Surgery, Biomedical Park Hospital, National Taiwan University Hospital, Hsinchu, Taiwan, ROC; 3 Division of Colorectal Surgery, Department of Surgery, National Taiwan University Hospital and College of Medicine, Taipei, Taiwan, ROC

**Keywords:** Medical research, Gastrointestinal diseases, Cancer, Colorectal cancer

## Abstract

The role of minimally invasive surgery (MIS) to treat clinically T4 (cT4) colorectal cancer (CRC) remains uncertain and deserves further investigation. A retrospective cohort study was conducted between September 2006 and March 2019 recruiting patients diagnosed as cT4 CRC and undergoing MIS at a university hospital and its branch. Patients’ demography, clinicopathology, surgical and oncological outcomes, and radicality were analyzed. A total of 128 patients were recruited with an average follow-up period of 33.8 months. The median time to soft diet was 6 days, and the median postoperative hospitalization periods was 11 days. The conversion and complication (Clavien–Dindo classification ≥ II) rates were 7.8% and 27.3%, respectively. The 30-day mortality was 0.78%. R0 resection rate was 92.2% for cT4M0 and 88.6% for pT4M0 patients. For cT4 CRC patients, the disease-free survival and 3-year overall survival were 86.1% and 86.8% for stage II, 54.1% and 57.9% for stage III, and 10.8% and 17.8% for stage IV. With acceptable conversion, complication and mortality rate, MIS may achieve satisfactory R0 resection rate and thus lead to good oncological outcomes for selected patients with cT4 CRC.

## Introduction

Minimally invasive surgery (MIS) is characterized by its more minimal physical incisions and faster recovery by utilizing advanced imaging systems and instruments compared with conventional open surgery. Nowadays, MIS, including laparoscopic or robotic surgery, has gained its significance and wide acceptance in surgical fields such as colorectal cancer (CRC) treatment. Laparoscopic colorectal resection is currently the mainstay treatment for the management of CRC. Robust evidence has shown the advantages of quicker postoperative convalescence and equivalent oncological outcomes using laparoscopic surgery compared to the open method^1–3^.

However, controversy remains when attempting to extend the indication of laparoscopic surgery for treating locally advanced CRC such as clinically T4 (cT4) stage tumors. For example, cT4 CRC requires multi-visceral en bloc resection to ensure cancer-free radicality. In this situation, R0 resection remains the key pursuit of curative treatment for CRC. Nonetheless, demanding techniques may compromise the feasibility of laparoscopic surgery to remove the locally advanced cancer. As a result, some surgeons believe the open method to be more suitable than laparoscopic surgery. Second, only limited studies have reported the short-term surgical and long-term oncological results for both laparoscopic and open surgeries^4,5^. Accordingly, the laparoscopic approach for colectomy is still not recommended for treating locally advanced CRC based on the National Comprehensive Cancer Network^®^ (NCCN^®^) guidelines^6,7^.

With the maturation of laparoscopic skills and the introduction of robotic surgery, the feasibility of MIS for cT4 CRC should be re-evaluated. The aim of this study was to evaluate the safety, feasibility, and efficacy of MIS for cT4 CRC in terms of surgical results and long-term oncological outcomes.

## Materials and methods

### Patient selection

One hundred and twenty eight patients diagnosed with cT4 CRC and underwent MIS (laparoscopic surgery and robotic surgery) in the National Taiwan University Hospital (NTUH), NTUH Yunlin Branch, and Hsinchu Branch, were recruited in this study between September 2006 and March 2019. The American Joint Committee on Cancer (AJCC) staging system 8th edition defines cT4 CRC as follows: (1) the tumor penetrates the visceral peritoneum (cT4a) or (2) the tumor invades the adjacent organ (cT4b). The diagnosis of cT4 CRC was established either preoperatively by imaging, such as a computed tomography (CT) scan, magnetic resonance imaging (MRI), whole body bone scan, or positron emission tomography (PET), or intraoperatively based on surgical findings such as grossly visceral serosa involvement or tumor adherence to the adjacent organ. This study recruited patients with cT4 CRC regardless of their nodal status.

MIS included laparoscopic and robotic surgery. The procedures of laparoscopic surgery were detailed previously^8^, and the robotic surgery was achieved with da Vinci robotic surgical systems. The settings and surgical procedures of the robotic surgery were reported in our previous articles^9^. Six laparoscopic surgeries were performed by Dr. YTL, and the remaining 122 surgeries (laparoscopic surgery and robotic surgery) were performed by Dr. JTL. The study was approved by the Institutional Review Board of NTUH, which waived the informed consent (201912125RINB). All methods were performed in accordance with the relevant guidelines and regulations.

For these 128 patients with cT4 CRC, concomitant cancerous conditions found preoperatively or intraoperatively included the following: (1) solely cT4 CRC (cT4a) or (2) cT4 CRC with adjacent organ involvement (cT4b) with or without (1) clinical peritoneal seeding (cM1c) and/or (2) resectable/unresectable distant metastasis, such as to the liver or lung (cM1a or cM1b). We defined surgery for cM1 patients as palliative surgery. For cM0 patients, “intention-to-treat” surgery was performed. In this study, “intention-to-treat” surgery indicated that the surgeons attempted to achieve R0 resection of the primary tumor for cM0 patients. A total of 7 cM1 patients underwent “intention-to-treat” surgery, indicating that the oligometastasis or localized peritoneal seeding of these patients was removed or ablated during the surgery. However, because of the small number of these patients (N = 7) in our study, we did not further analyze the oncological results of these patients.

R0 resection was defined as negative margin involvement on microscopic examination. R1 resection was defined as positive margin involvement on microscopic examination or microscopically < 1 mm of the resected margin. R2 resection was defined as (1) positive resected margin by the naked eye, (2) unresectable peritoneal seeding, or (3) distant metastasis.

All patients underwent colonoscopy and were diagnosed with cancer as confirmed by preoperative pathological examination. The preoperative evaluation of the CRC included historical information, physical examination, laboratory study, and imaging study. The laboratory study included complete blood cell count, differential count, and biochemistry examination such as liver or renal function, electrolytes, coagulation profiles, and carcinoembryonic antigen (CEA) levels. The imaging study included chest X-ray and chest/abdomen/pelvic CT scan with or without contrast (if the patients had contraindications to the intravenous contrast injection). Clinical staging was determined by CT scan, MRI, whole body bone scan, or whole PET scan by radiologists or determined by surgeons intraoperatively. The patients’ treatment plan was discussed in a multidisciplinary team in NTUH and its branch. The final diagnosis and staging of cancer were verified by the pathologists.

### Surveillance

All patients had regular follow-up that consisted of periodic physical examinations and blood chemistry panels (i.e. complete blood cell count and CEA) every 3–6 months. Additionally, they underwent colonoscopy, abdominal ultrasonography and chest/abdomen/pelvic CT scan or MRI every 6–12 months. Diagnoses of local recurrence or distant metastasis were based on colonoscopy or imaging studies.

### Statistical methods

Descriptive statistics were used to present the data, and for continuous parameters, percentage, frequency, medium, mean, standard deviation, and quartile were utilized, and frequency and percentage were used for categorized parameters. The disease-free survival (DFS) was measured from the date of the primary colonic surgery to the date of recurrence. The overall survival (OS) time was calculated from the date of surgery to the time of the last visit or death. Follow-up was updated on December 1, 2019. Survival was demonstrated using the Kaplan–Meier curve and analyzed using the log-rank test (Mantel–Cox) when comparing survival curves. A probability value of less than 0.05 was considered significant, and all statistical tests were two-sided. These analyses were performed using the Statistical Analysis System (SAS) version 9.4 for Windows.

## Results

### Demography and clinicopathology

**Figure 1:**
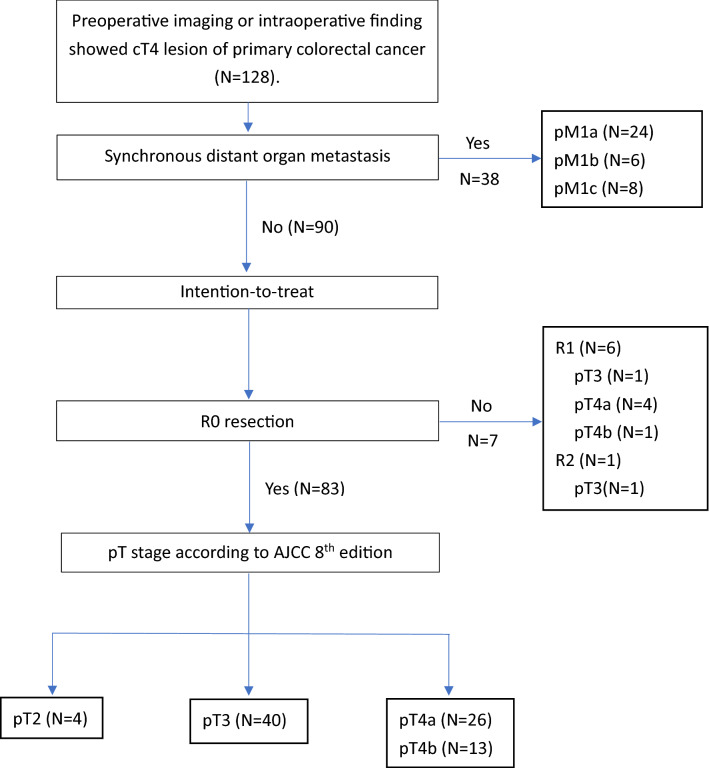
Patients’ recruitment and selection for clinically T4 lesion of colorectal cancer.

In total, 128 cT4 CRC patients underwent laparoscopic en bloc resection of primary CRC during the 13-year period. Patients’ demography and clinical parameters are listed in Table 1. The main primary tumor site was left-sided. Clinical metastatic lesions (distant metastasis or peritoneal carcinomatosis) accounted for 29.7% of cases, and ten patients underwent preoperative chemoradiation therapy. The flowchart of recruited patients is presented in Fig. 1.

**Table 1:** Demographic and clinical data of patients (N = 128).

Age (years) [median ± SD, (range)]	62.5 ± 13.8 (28–88)
Gender (female/male)	56/72
Body mass index (kg/m^2^) (mean ± SD)	23.5 ± 4.2
ASA (I/II/III/IV)	4/51/72/1
**Previous abdominal surgery**	
Open	6
Laparoscopy	3
**Location of cancer, n (%)**	
Cecum	2 (1.6%)
Ascending colon	23 (18.0%)
Hepatic flexure	2 (1.6%)
Transverse colon	4 (3.1%)
Splenic flexure	1 (0.8%)
Descending colon	11 (8.6%)
Sigmoid colon	41 (32.0%)
Rectosigmoid junction	19 (14.8%)
Rectum	25 (19.5%)
**Clinically N staging**	
N0	26
N1	42
N2	60
**Clinically M staging**	
M0	90 (70.3%)
M1	38 (29.7%)
Preoperative chemoradiation therapy	10
Preoperative CEA level (ng/mL), median (range)	23.6 (0.44–2167.3)

*BMI* body mass index, *ASA* American society of Anesthesiology.

### Surgical parameters

The surgical procedures of the primary CRC and the suspected organ involved by cT4 CRC undergoing the synchronous resection are shown in Table 2. In total, 113 patients underwent laparoscopic surgery and 15 patients underwent robotic surgery. cT4 CRC most frequently involved the bladder (N = 23), followed by the female adnexa (N = 16), and the small bowel (N = 13). Consequently, partial cystectomy (N = 21), oophorectomy (N = 18), and small bowel resection (N = 13) were the top three procedures performed. For two patients who underwent hysterectomy due to the involvement of the uterus, bilateral oophorectomy was resected prophylactically.

**Table 2:** Surgical procedures of the primary CRC and the suspected organ involved by cT4 CRC undergoing the synchronous resection (N = 128).

**Laparoscopic procedures (N = 113)**	
Right hemicolectomy	29
Extended right hemicolectomy	2
Left hemicolectomy	10
Extended left hemicolectomy	1
Anterior resection	22
Low anterior resection	46
Abdominoperineal resection	2
Subtotal colectomy	1
**Robotic method (N = 15)**	
Low anterior resection	12
Hartmann procedure	2
Abdominoperineal resection	1
**Clinically involved organs by cT4 CRC**^a^	
Small bowel	13
Duodenum	2
Urinary bladder	23
Uterus	8
Adnexa (unilateral/both)	8/8
Vagina	3
Proximal colon	1
Prostate	1
Seminal vesicle	1
Presacral area	1
Peritoneum	7
Pancreas	1
Omentum/mesentery	4
Gerota fascia	1
Ureter	1
Appendix	1
Abdominal wall	1

^a^A patient might have more than one organ involved.

Patients’ clinicopathological and molecular biological features are shown in Table 3. The median surgical time was 309 min with a median blood loss of 175 ml. Conversion from MIS to open surgery was required in 10 patients (7.8%).

**Table 3:** The clinico-pathological and molecular biologic features of patients.

Surgical time (min) (median, range)	309 (117–816)
Blood loss (ml) (median, range)	175 (30–1200)
**Diverting stoma [n (%)]**	
Colostomy	7 (5.5%)
Ileostomy	23 (18.0%)
Conversion to open method	10 (7.8%)
**Radicality of surgery [n (%)]**	
R0	83 (92.2%)
R1	6 (6.7%)
R2	1 (1.1%)
Tumor size (cm) (mean [range])	6.2 (2.2–18)
Harvested lymph nodes [median, IQR (25–75%)]	26 (17–34)
**Differentiation**^a^	
Well/moderately differentiated	113
Poorly/undifferentiated	13
**pT stage**	
T2	5
T3	50
T4a	47
T4b	26
**pN stage**	
N0	37
N1	45
N2	46
**pM stage**	
M1a	24
M1b	6
M1c	8
**TNM stage**	
II	35
III	55
IV	38
KRAS wide type/mutation^b^	73/33
BRAF wide type/mutation^c^	25/4
MMR deficiency/proficiency^d^	1/18

^a^The grade of differentiation was not reported by pathologists in 2 patients because of status post concomitant chemoradiation therapy.

^b^Twenty-two patient’s KRAS status were not available.

^c^Only 29 patients’ data were available.

^d^Only 19 patients’ data were available.

### Radicality

With regards to radicality, for 90 cT4M0 patients, 83 (92.2%) achieved R0 resection. Six patients (6.7%) achieved R1 resection, and 1 (1.1%) patient achieved R2 resection as demonstrated by pathological examination.

Seven patients with inadequate R0 resection (R1/R2 resections) are presented in Table 4. One patient with primary tumor involving the superior mesenteric vein trunk achieved R2 resection. One patient with primary tumor invading the left ureter and bladder underwent converted open anterior resection, partial bladder resection and left ureter segmentectomy. This patient had achieved R1 resection, as confirmed by the pathologist, because of margin involvement.

**Table 4:** Clinically M0 patients with R1 or R2 resection.

No	Age	Gender	Primary cancer site	Staging	Surgery^a^	cT4 description	Radicality	Description of incomplete radicality
1	76	Male	Ascending colon	pT3N2b	L. right hemicolectomy	Colon serosa involvement	R2	Superior mesenteric vein trunk involvement
2	49	Male	Middle rectum	pT4aN1c	L. LAR	Right-sided pelvic wall involvement	R1	Margin involved by carcinoma
3	56	Female	Rectosigmoid junction	pT4aN2a	L. LAR	Rectal serosa involvement	R1	Margin involved by carcinoma
4	59	Male	Rectosigmoid junction	pT3N1b	L. LAR	Rectal serosa involvement	R1	Margin involved by carcinoma
5	88	Male	Middle rectum	pT4bN2a	R. Hartmann procedure + partial bladder resection	Bladder wall invasion	R1	Radical margin involved by carcinoma
6	62	Female	Sigmoid colon	pT4bN2a	L.AR, conversion to open AR + partial bladder resection + ureter segmentectomy	Left ureter and bladder involvement	R1	Margin involved by carcinoma
7	63	Male	Low rectum	pT4aN0	R. LAR^b^	Presacral involvement	R1	Margin < 1 mm

^a^“L.” is the abbreviation of “laparoscopic”. LAR: low anterior resection. AR: anterior resection. “R.” is the abbreviation of “robotic”.

^b^The patient received preoperative neoadjuvant chemoradiation.

For 44 pT4M0 patients, 39 (88.6%) underwent R0 resection.

### Recovery and oncological results

Surgical outcomes of the patients are shown in Table 5. The median time to flatus was 4 days, and the time to soft diet was 6 days. The median number of postoperative hospitalization days was 11 (2–89) days. The numbers of complications using the Clavien–Dindo classification were 18 patients in class II and 17 patients in class III/IV. One patient (0.78%) died of pulmonary embolism 6 days after surgery. Anastomotic leakage remained the most common complication after colectomy, accounting for 10 patients.

**Table 5:** Surgical outcomes of patients.

Day to flatus (days) [median (range)]	4 (2–27)
Soft diet (days) [median (range)]	6 (3–29)
Postoperative hospitalization (days) [median (range)]	11 (2–89)
30 days mortality [n (%)]	1 (0.8%)
**Clavien–Dindo classification [n (%)]**	
II	18 (14.1%)
IIIa/IIIb	5/5 (7.8%)
IVa	6 (4.7%)
IVb	1 (0.8%)
**Complications**^a^	
Leakage	10
Ileus	1
Wound infection	2
Urinary tract infection	3
Urinary retention	1
Pulmonary emboli	1
Cerebrovascular accidence	1
SMV bleeding	1
Retroperitoneal abscess	1
Respiratory failure	1
Recto-vesicle fistula	1
Ureter injury	2
Pneumonia	4
Pelvic abscess	3
Recto-vaginal fistula	1
Internal herniation	1
Herpes zoster	1
Myocardial infarction	1
Adjuvant chemotherapy	109
Recurrence	52
Follow-up period (months) [mean (range)]	33.8 (0.2–159.0)

^a^A patient may have more than 1 type of complication.

**Figure 2:**
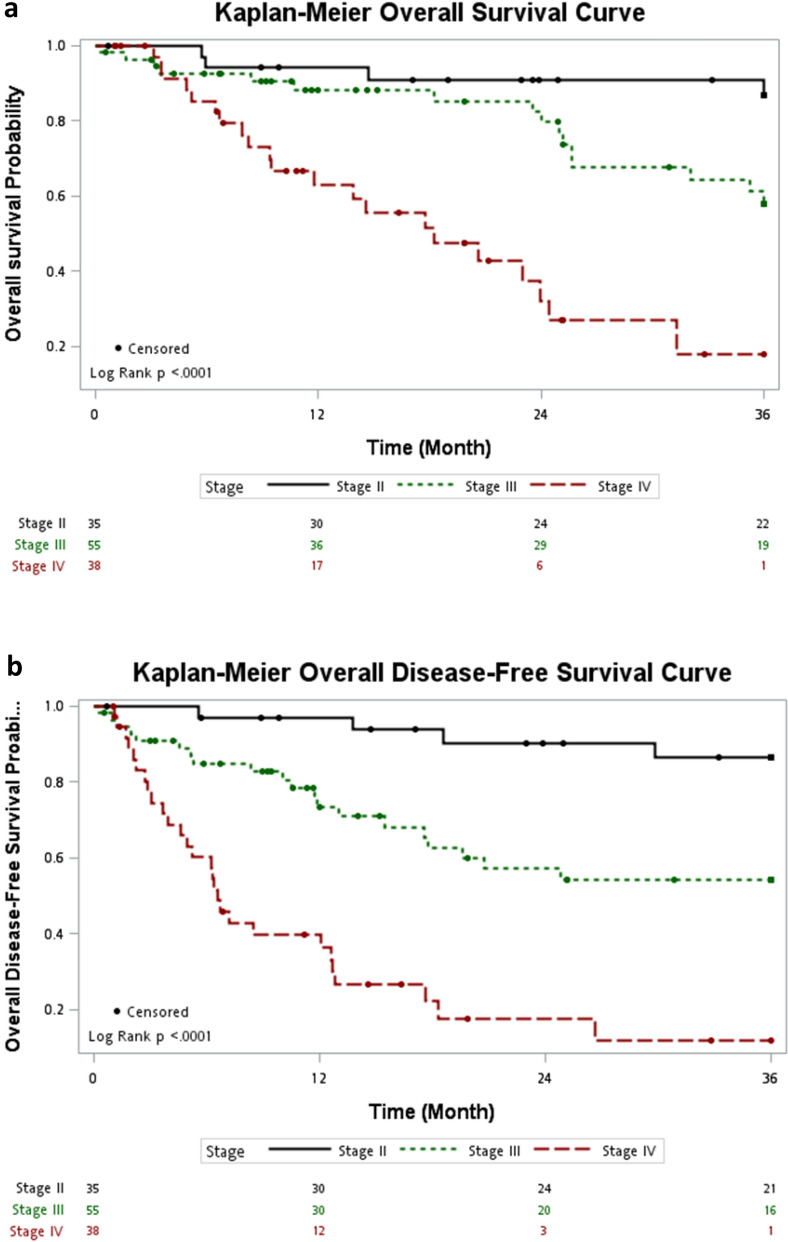
(**a**) Overall survival according to the tumor-node-metastasis (TNM) stages. (**b**) Disease-free survival according to the tumor-node-metastasis (TNM) stages.

In this study, the 3-year OS and DFS corresponded with the oncological pattern predicted by tumor-node-metastasis (TNM) staging (Fig. 2). The 3-year OS was 86.8% for stage II (95% confidence interval [CI] 0.725–0.964), 57.9% for stage III (95% CI 0.414–0.736), and 17.8% for stage IV (95% CI 0.036–0.398). The DFS was 86.1% for stage II (95% CI 0.670–0.946), 54.1% for stage III (95% CI 0.378–0.679), and 10.8% for stage IV (95% CI 0.190–0.287).

**Figure 3:**
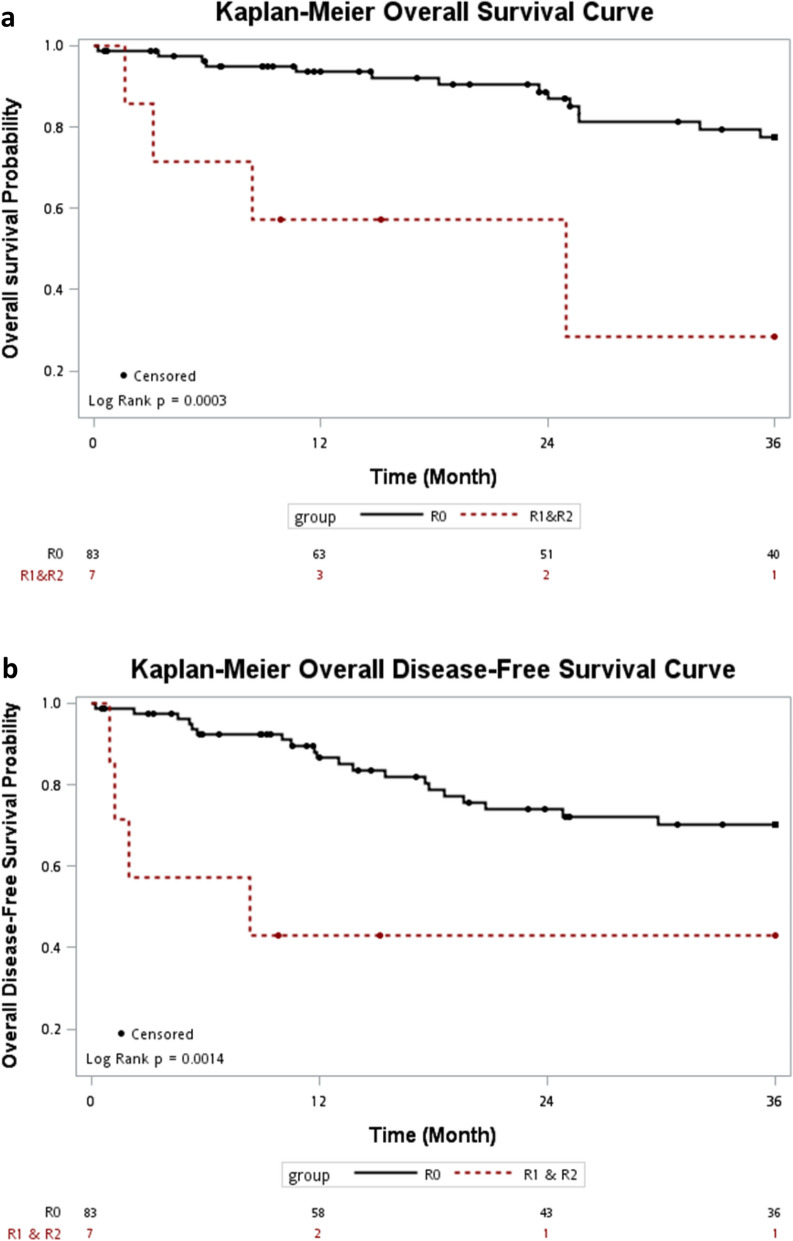
(**a**) Overall survival according to the radicality. (**b**) Disease-free survival according to the radicality.

With regards to the radicality (R0 vs. R1/R2), the 3-year OS and DFS were 77.6% (95% CI 0.663–0.872) and 70.3% (95% CI 0.588–0.807) for R0, and 28.6% (95% CI 0.007–0.750) and 42.9% (95% CI 0.113–0.781) for R1/R2, respectively. The patients of R0 resection had longer 3-year OS (*P* = 0.0003) and DFS (*P* = 0.0014) than non-R0 resection patients (Fig. 3).

We defined the right-sided colon as the cecum, ascending colon, hepatic flexure and proximal transverse colon, and the left-sided colon as the distal transverse colon, splenic flexure, descending colon, sigmoid colon, rectosigmoid junction and rectum. There was no survival difference including 3-year OS and DFS in terms of tumor sidedness (*P* = 0.0834 and *P* = 0.6543, respectively).

## Discussion

Our study showed that MIS could be a safe and feasible method for treating cT4 CRC. For these patients, as long as an R0 resection could be achieved, MIS could confer satisfactory DFS and OS. MIS could achieve similar conversion, complication and mortality rate to those of open surgery in previous reports. With a satisfactory R0 resection rate, MIS may achieve good oncological outcomes for selected patients with cT4 CRC.

Several studies have shown that laparoscopic surgery could achieve similar short-term surgical results and long-term oncological outcomes in treating pathological T4 (pT4) CRC with those of the open method^10–15^. However. in the real-world scenario, the decision on how surgeons choose to treat cT4 CRC is more complex. With the aim to achieve cancer clearance, i.e. R0 resection, any suspicious cancerous involvement should be removed. The procedure itself would be technically demanding because of the inability to avoid resection of additional organs in cases where the cancer adhered to the adjacent organ. Thus, the procedure to remove the suspicious cancerous lesion has the possibility to remove an organ that eventually had no cancer involvement. Moreover, the utilization of MIS might become formidable, even in the hands of experienced laparoscopic surgeons, and the requirement of concomitant organ resection might also result in a higher conversion rate. As a result, some surgeons may prefer laparotomy instead of the laparoscopic method in cT4 CRC cases^16^. In this context, we believe that recruiting cT4 CRC, rather than pT4 CRC may better reflect the real-world situation.

To our best knowledge, there are only limited studies that analyze the results of MIS for cT4 CRC. Part et al. recruited 71 laparoscopic and 222 open surgery patients with clinically suspected T4 CRC, and demonstrated that all the patients had pT4 CRC with similar perioperative and long-term oncological outcomes^16^. Furthermore, Huh et al. compared 24 laparoscopic and 19 open surgery patients, and came to a similar conclusion that short-term outcomes were similar between two groups. About 33.3% (8/24 patients) was pT4 CRC. In our study, 44 (34.4%) and 29 (22.7%) patients were diagnosed with pT4M0 and pT4M1 CRC, respectively.

The conversion rate in previous studies was approximately 10.7% (range 7.1–28.2%)^17^, and in the current study, 10 patients (7.8%) required open conversion. Among these patients, except in four patients who underwent palliative surgery, R0 and R1 resections were achieved in 5 and 1 patients, respectively. The patient who achieved R1 resection was due to left ureter and bladder invasion. Conversion should be deemed as an alternative treatment modality to achieve R0 resection, rather than a failure of surgery.

With regards to patients without distant metastasis or peritoneal carcinomatosis, intra-abdominal organ involvement was not contraindicated to perform MIS^18^. The combined procedures such as partial bladder resection, small bowel resection, or hysterectomy could be safely performed by an experienced surgeon. Again, we believe that the surgical method must be tailored to the severity of the cancerous invasion, as well as the surgeon’s experience. Moreover, the decision to perform MIS should never compromise an R0 resection.

The complication rate classified by the Clavien–Dindo classification ≥ II was 27.3% in our study; this is slightly higher than those reported in previous studies (7.3^10^–16.9%^13^). The higher complication rate in the current study might be due to the larger numbers of Clavien–Dindo classification II patients in our series. Indeed, the complication rate decreased to 13.3% if we only considered Clavien–Dindo classification III/IV patients, which is similar to those of the same severity reported in previous studies (1.5^10^–12.2%^13^). Anastomotic leakage remained the major complication; this accounted for 7.8% in the current study, corresponding to the reported rate of 3^14^–10%^19^.

The post-operative recovery was tolerable in our study in terms of the first day of flatus, day of resuming soft diet intake, and day of post-operative hospitalization^10,11,14,20^. However, given that a universal post-operative protocol for cT4 CRC patients after MIS is lacking, it was difficult to compare the recovery results between studies.

The R0 rate in the current study was 92.9% for cT4 CRC, corresponded to those reported in previous studies (75–94%^10,13,21^). The R1 rate was 6.7% in our study, while those reported by Angelis et al. and Leon et al. were 5.7% and 11.8%, respectively. Similarly, the R0 rate for pT4 CRC in our study was 88.6%, corresponded to those reported in previous studies^5^.

Our study showed that patients with R0 resection had more favorable outcomes, both in terms of DFS and 3-year OS, when compared to those with non-R0 resection. Although the case numbers of non-R0 were small (N = 7), we found that the *P*-value was relatively small, indicating that the survival difference is less likely due to “chance” and could be reliable. Again, the results of our study supported the notion that R0 resection remains the central pursuit of curative surgery. MIS should not compromise this principle.

Although surgery for cM1 CRC patients is considered a palliative treatment, some studies have made significant effort to perform “intention-to-treat” treatment for cM1 CRC patients. The “intention-to-treat” principle indicated the removal of all grossly suspicious or evident metastatic lesions with ablation, radiotherapy or surgical method. Several previous studies have revealed the oncological benefits of debulking surgery for isolated pulmonary/hepatic metastases or abdominal nodal recurrence^22,23^. Furthermore, Johnson et al. reported that a curative-intent trimodality approach could provide favorable survival in selected metastatic CRC patients with isolated retroperitoneal or mesenteric nodal recurrence. The curative-intent trimodality approach combined the external beam radiotherapy with chemotherapy, radical surgery and intraoperative radiotherapy^24^. In our study, seven pM1 patients underwent the “intention-to-treat” procedure. However, we did not attempt to analyze the DFS and 3-year OS of these patients because of smaller patient numbers.

Localized peritoneal carcinomatosis, although categorized as pM1c, is a special situation that required surgeons’ continuous effort to remove all visible tumors if technically feasible. Hyperthermic intraperitoneal chemoperfusion (HIPEC) has been introduced in recent decades; however, only two patients in our series received HIPEC, which was too small to draw any conclusions. Nevertheless, we believed that pT1c, specifically localized peritoneal carcinomatosis, remains a field worthy of investigation with the purpose to achieve curative intent.

Our study has several limitations. First, this was a one-arm study with no comparison group. The lack of an open control group was due to the fact that MIS is the standard surgical procedure for treating CRC in our institute. As a result, we intended to compare our results with those in previous studies to overcome this limitation. Second, this study recruited patients from a 13-year period, and there have been considerable improvements in survival over this period due to the introduction of new therapeutic modalities such as new chemotherapy regimens and targeted therapy. Similarly, concomitant chemoradiation therapy, currently a standard treatment of locally advanced rectal cancer, was administered in 10 patients (41.7%) with locally advanced rectal cancer. The heterogeneity of patient composites may result in potential bias. Third, several molecular markers such as Kirsten rat sarcoma viral oncogene homolog (KRAS), v-raf murine sarcoma viral oncogene homolog B1 (BRAF), or mismatch repair (MMR) status have been discovered recently. Our study failed to classify CRC by these markers.

## Conclusion

The present study suggested that patients with cT4 CRC undergoing MIS might achieve good recovery and similar conversion, complication and mortality rate to those of open surgery reported in previous studies. MIS could lead to a satisfactory R0 resection rate, and therefore achieve satisfactory oncological outcomes for selected patients with cT4 CRC.
